# A Review of Histological Techniques for Differentiating Human Bone from Animal Bone

**DOI:** 10.3390/mps7040051

**Published:** 2024-06-30

**Authors:** Emanuela Stan, Camelia-Oana Muresan, Ecaterina Daescu, Raluca Dumache, Veronica Ciocan, Stefania Ungureanu, Dan Costachescu, Alexandra Enache

**Affiliations:** 1 Department of Neuroscience, Discipline of Forensic Medicine, Bioethics, Deontology and Medical Law, “Victor Babes” University of Medicine and Pharmacy, 300041 Timisoara, Romania; emanuela.stan@umft.ro (E.S.); muresan.camelia@umft.ro (C.-O.M.); raluca.dumache@umft.ro (R.D.); veronica.luta@umft.ro (V.C.); stefania.ungureanu@umft.ro (S.U.); enache.alexandra@umft.ro (A.E.); 2 Institute of Legal Medicine Timisoara, 300610 Timisoara, Romania; 3 Ethics and Human Identification Research Center, Department of Neurosciences, “Victor Babes” University of Medicine and Pharmacy, 300041 Timisoara, Romania; 4 Department I of Anatomy and Embryology, “Victor Babes” University of Medicine and Pharmacy, 300041 Timisoara, Romania; 5 Radiology Laboratory, Emergency Municipal Clinical Hospital Timisoara, 300254 Timisoara, Romania; costachescu.dan@umft.ro; 6 Department of Orthopedics-Traumatology, Urology, Radiology and Medical Imaging, Discipline of Radiology and Medical Imaging, “Victor Babes” University of Medicine and Pharmacy, 300041 Timisoara, Romania

**Keywords:** human bone, non-human bone, histomorphometric methods, Haversian systems

## Abstract

The first step in anthropological study is the positive identification of human remains, which can be a challenging undertaking when bones are broken. When bone pieces from different species are mixed together, it can be crucial to distinguish between them in forensic and archaeological contexts. For years, anthropology and archaeology have employed the histomorphological analysis of bones to evaluate species-specific variations. Based on variations in the dimensions and configuration of Haversian systems between the two groups, these techniques have been devised to distinguish between non-human and human bones. All of those techniques concentrate on a very particular kind of bone, zone, and segment. Histomorphometric techniques make the assumption that there are size, form, and quantity variations between non-humans and humans. The structural components of Haversian bones are significant enough to use discriminant function analysis to separate one from the other. This review proposes a comprehensive literature analysis of the various strategies or techniques available for distinguishing human from non-human bones to demonstrate that histomorphological analysis is the most effective method to be used in the case of inadequate or compromised samples.

## 1. Introduction

Forensic anthropologists frequently struggle with the identification of fragmented skeletal remains. Determining whether a bone is human or non-human is a crucial topic that must be addressed from the outset and will have a significant impact on the course of the criminal inquiry. Skeletal fragments may be purposefully or unintentionally mixed with animal bones during major disasters and domestic crimes, making identification difficult [1,2]. Therefore, it is essential to identify each fragment, and anthropologists look for the quickest, least expensive, and most trustworthy way to do so. 

Since the 1800s, anthropology and archaeology have employed the histomorphological analysis of bones to evaluate species-specific characteristics. To comprehend the appearance and measure tissue features, histomorphology entails viewing thin transverse slices of cortical bone under a magnification score of 100–200× [1]. The ability to distinguish between human and non-human bone and between non-human species is another important function of bone histology [3,4,5]. When bone fragments from different species are mixed together, it can be crucial to distinguish between them in forensic and archaeological contexts. The identification of disaster victims and forensic archaeological scenarios, including mass grave sites, both contemporary and historical, requires the establishment of a minimum number of human beings in order to assist eventual identification, which makes this particularly crucial [6]. 

Histology is a frequently utilized identification method when macroscopic anatomical characteristics are altered or absent or can be used as an auxiliary tool for macroscopic observation, as numerous investigations have shown that human and non-human bone microstructures differ noticeably [7]. Histology serves as a valuable adjunct to macroscopic observation in various scientific disciplines, including palaeontology [8]. While macroscopic examination provides initial insights into bone morphology and general characteristics, histological analysis offers a deeper understanding of bone structure at a microscopic level. This microscopic perspective, particularly in accurate species identification, can reveal details that are crucial for the assessment of bone growth patterns, and inference of biological traits [9]. Histological determination has been carried out using two alternative methods. First, the examination of bone organization and traits thought to be unique to human or non-human microstructures forms the foundation of the qualitative method [1]. Plexiform, also known as the fibrolamellar complex, is a fast-growing main organization present in the majority of non-human animals. As plexiform can be present in human bone when there has been a repair or in juvenile bone, it is therefore believed to be a reliable sign of non-human bone [10]. Mulhern et al. [11] have identified other structures, such as osteon banding, as reliable indicators of non-human origin. However, recent research [12] has shown that human bone contains osteon banding. Haversian bone, also known as a secondary osteon, is a type of bone that develops in cortical bone and is distinguished by the replacement of pre-existing bone with new bone through structures known as Haversian systems [5]. Plexiform and Haversian bones can coexist in most mammals and some birds. The latter is typically found near the periosteal surface in large animals, while the former is primarily found near the endosteal surface [13]. Dense or dispersed secondary osteons are seen in human bone tissue. Nevertheless, due to its existence in other mammals, Haversian bone by itself is unable to confirm a human origin [5]. The quantitative method, also known as the histomorphometric method, was created to distinguish between Haversian bone from non-human sources and human bone. The underlying premise of histomorphometric approaches is that the amount, shape, and size differences between Haversian bone structural features in humans and non-humans are significant enough to be distinguished using discriminant function analysis. When a qualitative signal, such as plexiform bone, is absent, those techniques are more crucial. Large mammal bones may exhibit this, with the endosteal region filled with Haversian bone remaining the only intact portion after the subperiosteal surface is destroyed by weathering, fire, severe fragmentation, or other peri- or post-mortem change [14].

The differentiation between human and animal bones is critical to forensic and archaeological investigations. While macroscopic morphological analysis is often the first step in identifying skeletal remains, there are numerous instances where this method alone is inadequate. Macroscopic features may not be discernible in cases involving highly fragmented, weathered, or otherwise compromised bones. This is where histological analysis becomes indispensable. Histological methods provide a microscopic examination of bone tissue, revealing structural details not visible to the naked eye. These methods are particularly useful when skeletal fragments are too small or degraded to exhibit apparent morphological features, the remains are heavily burned or otherwise altered by environmental conditions, or differentiation between human and animal bones is necessary for legal or forensic purposes where precise identification is required [14]. This being said, one of the first and most crucial stages of forensic research is the identification of the material being studied. This may be the discrimination of human remains from those of other primates or the preparation of bone artifacts from stone. It is at this juncture that histological techniques can play a vital role in the analysis of material that is often poorly preserved and difficult to identify through gross morphology. 

This review aims to synthesize the current literature on histological methods for differentiating human from animal bones, focusing on the microscopic features of the most common species found in our country. It is important to be able to differentiate between human and non-human bone, as forensic investigation often involves the identification of victim remains, human or non-human.

## 2. Human Bone vs. Non-Human Bone

It has been helpful to employ conventional 2D histology techniques to determine species origin in situations where skeletal remains are typically extremely fragmented, deteriorated, burned, or otherwise unidentified. 

The primary method for qualitatively analysing cortical bone is to identify the type, pattern, and organization of bone tissue. Mammalian bone, for instance, can have Haversian, fibrolamellar, lamellar, or woven bone tissue types [15]. Circumferential lamellar layers can be used to identify laminar fibrolamellar bone. Intermittent sheets of woven and lamellar bone differentiate plexiform bone, an arrangement of fibrolamellar bone that resembles the design of bricks and is frequently observed in non-human animal bone. In adult human bone, this plexiform-type configuration of fibrolamellar bone is not frequently seen [16,17,18]. 

It is more challenging to differentiate Haversian bone, or secondary osteonal bone, between non-human and human species. Arrays of lamellar bone tissue with a central Haversian (vascular) channel are referred to as this type of bone. The characteristic hallmark of secondary osteonal bone tissue that defines the concentric lamellae is a reversal line. Numerous avian, mammalian, and reptilian species—including humans—have been reported to possess haversian bone [19]. Therefore, Haversian bone by itself cannot be used to diagnose human bone. Approaches for distinguishing human from non-human bone that depend on distinctions in Haversian canal size and osteon size and shape have been proposed but applied with different degrees of success. Therefore, it is important to use caution when using these strategies, as most of them remain to be validated [20].

### 2.1. Histological Structures 

The simplest anatomic unit of an osteon is a single Haversian system, which in the transverse section consists of a Haversian canal and its surrounding lamellae. Haversian systems are primarily longitudinal in human remains, with secondary osteons present only in areas where there has been remodelling during growth or at sites deep in the periosteal surface, such as in response to fractures or other bone injuries [21]. Primary bone is never completely resorbed during the process of remodelling; therefore, in dry human bone with little or no organic content, the mineralized structure of the bone, including the Haversian systems, is usually preserved. In contrast, the cortical bones of certain non-human species, such as sheep, can be completely remodelled without any primary bone tissue being left behind. In mature sheep ribs, the plexiform tissue is replaced by Haversian tissue, which consists of medium-sized and irregularly shaped canals. On the other hand, immature sheep femora consist of plexiform bone throughout, with a possibility of a few scattered Haversian systems located posteriorly [1,13]. This can present difficulties in differentiating human bones from those of these species using histological methods, as the absence of primary bone tissue in certain non-human species complicates the distinction [22].

#### 2.1.1. Osteon Structure

Osteons are the functional units of compact bone and represent a major advancement in the structural complexity of bone in vertebrates. They are primarily built to withstand the compressive forces on the bone. Osteons are comprised of a central (Haversian) canal that houses the blood and nerve supply for the osteon, the enclosed concentric lamellae of bone around this, and the osteocytes within small spaces (lacunae) between the lamellae [21,23]. The diameter and arrangement of osteons depend on the type of bone and the forces it has to withstand. This is important as it means that there are a variety of osteon patterns between human and different species of animal bone, and this variation can be used to differentiate between human and non-human bone [21,22]. In describing these differences, we can start to distinguish some histological techniques that separate human and non-human bones. The central canal of human osteons is approximately 50 micrometers in diameter, and the osteocytes surrounding it are arranged in a single file. In non-human bones of modern species, this canal is larger, and the osteocytes may be more haphazardly arranged. A resin cast of the canal could be observed under a microscope, and the size and pattern of the canal and arranged osteocytes may be compared to human and non-human standards [24]. This method has been previously used to distinguish between cow and red deer bones. High-quality histological sections of a human and non-human osteon could also be compared to each other, but this may prove difficult to obtain due to the destruction of the bone [23].

#### 2.1.2. Lacunae and Canaliculi

The bone matrix is laid down in lamellae. Each new lamella is added in such a way that it completely encircles a Haversian canal. This produces a series of nested cylinders of bone. Osteons are usually parallel to the diaphysis of the bone [24]. The osteocytes residing in the lacuna are interconnected to the rest of the bone and the blood supply by small channels. These structures are known as canaliculi. Osteons are not usually found in woven bone, the predecessor to lamellar bone, in which the collagen fibres are arranged haphazardly. Instead, there are just groups of osteocytes and irregularly distributed canals. The familiar cavities in bone tissue are referred to as lacunae. The osteocytes, which are bone cells with a spider-like form, are found within these structures. Within the osseous structure, there exists a distinct region surrounding the lacuna [25,26]. The phenomenon is referred to as a halo, which arises due to the space between the cell and the lacuna. Fixatives and stains usually wash this away, hence it is a feature of living bone. Osteocytes are not usually distributed evenly between the lacunae, and osteocyte count can be used as a rough indicator of bone health and the strains to which a bone has been subjected. Eroding bone diseases such as osteoporosis can be determined through a reduced osteocyte count [27,28].

#### 2.1.3. Remodelling Patterns

Remodelling is the process by which bones are renewed and repaired, maintaining the calcium homeostasis necessary for bodily function. It is a process that humans share with all other vertebrates, although the speed and efficacy of bone remodelling vary between species and are affected by factors such as body size, metabolic rate, and the mechanical function of the skeleton. During remodelling, bone is removed by osteoclasts, and new bone is formed by osteoblasts. At any time in the skeleton, there are two basic types of remodelling: surface remodelling, where the centre of a piece of bone is unchanged, and modelling, where bone shape and form are changed [24,29]. 

Primary osteons are formed in dense bone and are ultimately reconstructed into secondary osteons by a process of osteoclastic resorption and osteoblastic reformation. Primary osteons consist of a Haversian canal surrounded by concentric rings of lamella. Osteocytes are trapped in lacunae between lamella. Secondary osteons are formed in a similar fashion to primary osteons; however, they are typical of vascular bone and remodelling. As opposed to the Haversian canal, there are several canals within secondary osteons, and these are interconnected by a system of canaliculi. The lacunae in secondary osteons are elongate and more regularly aligned. Both types of osteons become secondarily mineralized from the centre outward. The canals and lacunae may contain unmineralized osteoid [24,30].

Several histological features define the process of bone remodelling in human bones. The remodelling of bone differs between human and animal bone, and features that are present in the bone as a result of the remodelling process sometimes remain long after the individual whose bone it is has died. Lamellar organization is one of the basic features of bone. It is formed by many thin layers of bone that are laid down over a long period of time. The presence of primary and secondary osteons is specific to remodelling in humans, as is evidence of the reversal phase of remodelling. Fibrolamellar bone has been identified as a distinct type of bone tissue that is only present following a fracture during the repair phase of remodelling. These features in bone resulting from remodelling are viable criteria for differentiating human bone from that of other species [29,31]. 

#### 2.1.4. Cement Lines

Cement lines, which are present in all bones, human or non-human, serve as a transient point of arrest in modelling, separating areas of bone formed at different times. The formation of bone cement lines can occur in two distinct ways. In primary bone, whether woven or lamellar, a cement line is left when a Haversian or remodelling space is filled with new bone. This occurs at the surface of the old bone. On the other hand, secondary bone only has an incremental line left where a packet of bone has been remodelled [5,32]. This occurs in two locations: at the edge of the new surface, which is formed by either a resorption cavity or a modelling surface, and also at a distance from the site of remodelling.

This evidence points towards cement line nature being similar in human and non-human bone, with the only differences occurring in the rate and quality of formation. This is a significant finding because a poor understanding of cement line nature in non-human bone could lead to difficulty in separating this from fragmentary or poorly preserved human material. This could result in inaccurate conclusions about whether bone is human or animal. Despite findings supporting a similar cement line nature in both types of bone, there are still some that argue cement lines demonstrate different characteristics in human and animal bone. It is suggested that the nature of cement line formation in relation to osteon size and shape will cause there to be differences in the cement line patterns of human and non-human material [23,33].

#### 2.1.5. Haversian System Variations

There are a wide range of Haversian system variations in humans and animals that affect the osteon shape and arrangement. The majority of the Haversian systems found in human compact bones are simple in form. This means they have only a few secondary osteons connected to a larger primary osteon, and they are dispersed evenly throughout the bone. However, there are a few locations in the human skeleton where the Haversian systems become more complex. In general, these occur in areas of bone that have been subjected to repeated microtrauma or areas of bone that have formed in response to mechanical stress. This bone will have a higher osteon packing density, with numerous interconnecting osteons to help resist the load. It will also have larger secondary osteons formed from the remodelling of existing primary osteons [34]. This is especially true in subperiosteal osteons formed in infants, which are remodelled to adult size and shape in the adolescent years. In contrast, animal compact bone can have a wide variety of Haversian system patterns, even within the same species. This depends on the species, breed, age, and even sex of the animal, and it is related to the specific bone function and the rate at which bone is renewed. This makes it extremely difficult to differentiate between animal and human bones and to identify the species and sometimes the sex of the animal from a histological study. Although there are exceptions, a general pattern is that animals have a lower osteon packing density with less interconnection and a greater range in both osteon and pore size than human bone. Young animals have a more irregular distribution of simple and compound primary osteons, with larger primary osteons away from the periosteum and simple osteons near the endosteum. This contrasts with regions of fast human bone formation in which primary osteons are uniformly small in size [1,4,35]. In the case of avian bone, generally there are no true Haversian systems or the Haversian systems are irregular, as all bone is remodelled through medullary expansion and the deposition of new bone at the periosteum. This makes it quite different from other types of bone, and the study of avian microanatomy is usually concerned with determining the rate of growth and age of the bird [36]. 

The bone matrix consists mainly of type I collagen fibres (approximately 90%) [26]. Bone matrices exhibit various patterns, including woven-fibered, parallel-fibered, or lamellar collagen fibre patterns [37]. Woven bone is characterized by irregular bundles of collagen fibres that are disorganized and loosely packed, forming a random matrix that appears woven. In parallel-fibered bone, collagen fibres are closely packed and aligned parallel to each other in large swathes, appearing smooth in cross-sections. In the lamellar bone matrix, collagen fibre orientations typically alternate between successive lamellae, leading to a characteristic striped alternation of light and dark lamellae in polarized light. This fibre organization allows the highest density of collagen per unit tissue volume [17,38]. 

Variations in collagen fibre orientation within the bone tissue matrix may represent biomechanical adaptations related to specific strain modes of tension, compression, or shear. Studies of variation in the orientation of collagen fibres have revealed that the orientation of collagen fibres within bones is not random but preferentially aligned to accommodate different loads. For instance, regions habitually loaded in compression have relatively more oblique-to-transverse collagen than regions loaded in tension, which are best resisted by fibres aligned longitudinally relative to the load [38,39,40,41,42,43]. 

In human bone osteons, collagen fibres are highly organized in a concentric lamellar pattern. Each lamella within the osteon has collagen fibres aligned at varying angles, creating a helicoidal or “twisted plywood” structure. Within each lamella, collagen fibres run parallel to each other but are oriented at different angles relative to adjacent lamellae. This alternating orientation gives osteons enhanced resistance to torsional (twisting) forces. The precise orientation of collagen fibres in osteons contributes to the bone’s ability to resist and distribute mechanical loads efficiently [23,40,44]. 

Similar to human bones, non-human bones also exhibit a concentric lamellar structure within osteons. However, the degree of collagen fibre orientation and the angles between layers can vary depending on the species. This variation in collagen orientation reflects adaptations to different lifestyles and environmental challenges [45,46]. For instance, animals that experience high torsional forces, such as those that run or jump frequently, may have osteons with collagen orientations optimized for those specific mechanical demands [39,47]. In a comprehensive study, Warshaw et al. [38] examined patterns of collagen fibre orientation in cross-sections from the midshaft femur, humerus, tibia, radius, and ulna in a wide array of living primate taxa. These taxa spanned from strepsirrhine families and platyrrhine families to the family Tarsiidae, encompassing a rich diversity of body sizes and positional behaviours. Their findings, which unveiled a preponderance of longitudinally oriented collagen in both periosteal primary and intracortically remodelled bone, provide a nuanced understanding of the complex adaptations in these species.

### 2.2. Histomorphometric Approach for Quantification of Microstructure in Human and Non-Human Species

The quantitative method, also known as the histomorphometric method, originated to distinguish Haversian bone from non-human sources and human bone. The underlying premise of histomorphometric approaches is that the amount, shape, and size differences between Haversian bone structural features in humans and non-humans are significant enough to be distinguished using discriminant function analysis [34].

According to Hillier et al. [1] and Lagace et al. [47], histomorphometric methods presume that the variations in the size, form, and amount of Haversian bone structural elements between humans and non-humans are significant enough to differentiate between them using discriminant function analysis. These techniques are particularly crucial in cases such as plexiform bone, where no qualitative signal is provided. This may be the situation with big mammal bones where the endosteal area filled with Haversian bone is the sole element remaining after the subperiosteal surface is eliminated by weathering, fire, severe fragmentation, or other peri- or post-mortem change [48]. 

Cattaneo and colleagues published a study in 1999 testing histological, immunological, and DNA approaches for human and non-human identification. The work presented here was the first to suggest using a discriminant function analysis to categorize bones into human and non-human categories. Three histomorphometric criteria for the Haversian canal are used in their method, which is based on long bones burned at temperatures between 800 and 1200 degrees Celsius. Because it involves a variety of animals (humans, horses, cows, sheep, swine, cats, and dogs), this method is very pertinent [7]. 

In 2006, Martiniaková and colleagues put forth a classification strategy for humans and four more species. They employ the mid-shaft femora segment from pigs, sheep, cattle, and rabbits in their technique. As part of their method for grouping things, they suggested seven histomorphometric parameters based on healthy osteons and Haversian canals [3]. 

Additionally, in 2012, Crescimanno and Stout released a method for distinguishing between humans and non-humans. Osteon circularity is the sole histomorphometric criterion that underpins their methodology. Their technique uses the humerus, femur, and ribs of humans as well as those of three other species (dogs, pigs, and deer) [49]. This method opens new doors for both archaeological and forensic purposes, providing a valuable tool for accurately identifying bone remains in various contexts. 

In 2012, Dominguez and Crowder presented an approach based on the area and circularity of undamaged secondary osteons. Their approach was centred on the long bones (femur and humerus) and flat bones (ribs) of three species: humans, deer, and dogs. In addition to a discriminant function for humans and non-humans, they developed a categorization function for the three species [50].

In a study conducted by Pirock et al. in 1996, major differences were observed between human ribs and femora with respect to the normal microstructural elements of bone. The study reported that the diameter of the Haversian system in ribs (n = 63) was 192 mm, whereas it was 270 +/− 35 mm for the femora (n = 4). For the ribs, the reported value for the diameter of the Haversian canal was 46 mm and for the femora, it was 60 +/− 34 mm [51]. In another study by Pfeiffer, significant differences (*p* < 0.01) were reported for the Haversian system area between ribs and femora in skeletal populations from 18th- and 19th-century cemeteries from Canada and Great Britain, and a 20th-century cadaver population from South Africa. The average Haversian system area measured 0.030 +/− 0.015 mm^2^ for the ribs (n = 100) and 0.041 +/− 0.021 mm^2^ for the femora (n = 41) [52]. 

Similarly, different bones within an individual non-human mammal may appear different histologically. 

In a study on cortical bone organization and its relationship with antemortem microdamage in 11 Rocky Mountain mule deer, Skedros et al. reported a general proximal-to-distal increase in the number of Haversian systems per square millimeter in the forelimb: humerus 2.45 +/− 1.90/mm^2^; radius 8.65 +/− 2.94 mm^2^; metacarpal 5.93 +/− 2.51 mm^2^; and phalanx 10.17 +/− 2.23 mm^2^ [53].

Analysis is necessary because a number of extrinsic factors, including age and sex, can cause variations in bone histomorphometry. Since the year 1965, several age-at-death estimation techniques have been developed using histomorphometry in human bone due to the well-established link between age and bone microstructure [54]. Although aging changes the size and form of Haversian systems, it additionally boosts their number [55]. This holds true for non-human bones as well. Although the impact of sex on histomorphometry and bone remodelling is more debatable, it does appear to matter. There was no correlation between sex and the histomorphometric factors examined in certain research [56]. The only approach among the four examined in the literature that examined the connection between sex and histomorphometric characteristics in the human sample was that of Crescimanno and Stout (2012). There was no statistically significant difference between males and females, according to these authors [49]. To build effective methods for species identification, a deeper comprehension of those characteristics as well as the typical variation in bone found in both humans and other animals is required. As a result, techniques should be based on characteristics that are less influenced by outside variables, like age or sex in both groups, in addition to strong discriminative variables for humans and non-humans. Table 1 summarises the main findings of the most important morphometric studies.

The study conducted by Morales et al. [57] analysed tibia bone sections from various species, including humans, bovines, pigs, hens, cats, and dogs. The samples were processed using conventional histological techniques and were observed under a microscope with a 40× magnification. The study examined the Haversian canal density parameters by mm^2^, the Haversian canal’s diameter, and the Haversian system’s diameter. The study used one-way ANOVA with a Scheff post-test, *p* < 0.05, to compare the parameters. The study’s results have significant implications for our understanding of bone structure. The human bone, for instance, exhibited a Haversian canal diameter that was notably different from that of cats and dogs, but not from pigs, bovines, and hens. Similarly, the osteon diameter in human bone was significantly distinct from cats, dogs, and hens, but not from pigs and bovines. Furthermore, the Haversian canals per mm^2^ in human bones showed marked differences from those in dogs, pigs, bovines, and hens, but not from those in cats. 

After comparing the Haversian canal density per mm^2^ variable, the mean difference between humans and bovines was 2.57, with a significance value of 0.023. Comparing the values obtained for the diameter of the Haversian canal variable, the mean difference between humans and dogs was 23.55, with a significance level of 0.002. In terms of the osteon diameter variable, the mean difference between humans and dogs was found to be 51.68 with a significance value of 0.000. When comparing the values obtained for the Haversian canal density per mm^2^ variable, the mean difference between humans and dogs was −2.52 with a significance value of 0.039. After comparing the Haversian canal density per mm^2^ between humans and pigs, a mean difference of 4.25 was found with a significance of 0.000. Comparing the values obtained for the osteon diameter and Haversian canal density per mm^2^ variables, there were significant mean differences between humans and hens (osteon diameter: mean difference of 65.33, sig. 0.000; Haversian canal density per mm^2^: mean difference of −13.13, sig. 0.000). When comparing Haversian canal and osteon diameters, the mean differences between humans and cats were 25.32* (sig. 0.001) and 61.14* (sig. 0.000), respectively.

**Figure 1:**
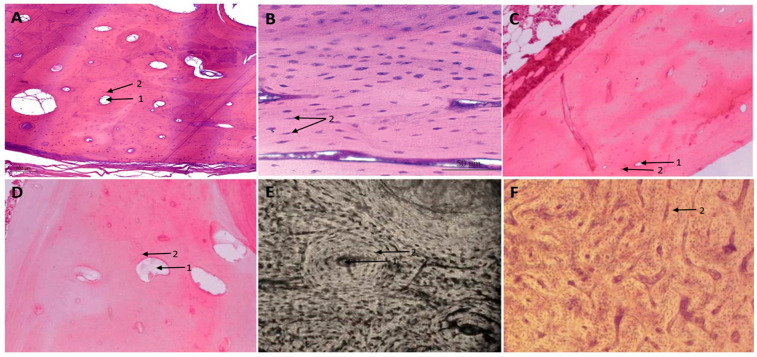
(**A**) *Homo sapiens* bone structure: compact osteons with a central canal (1) delimited by the presence of well-defined cement lines with the presence of osteocytes (2) in their corresponding lacunas (original image). (**B**) *Gallus gallus domesticus* (hen) longitudinal bone structure: small size osteons with spindle-shaped osteocyte lacunae (2) (image adapted from Domenis et al.) [66]. (**C**) *Felis catus* (domestic cat) bone structure: medium size osteons with a central canal (1) and osteocytes (2), with a great number of interstitial systems (image adapted from Morales et al. [57]). (**D**) *Canis lupus familiaris* bone structure: a high density of medium size osteons, with a central canal (1) and osteocytes (2), and a great number of interstitial systems (image adapted from Morales et al. [57]). (**E**) *Bos taurus* bone structure: dense Haversian tissue with a big osteon with a central canal (1) and osteocytes (2) (image adapted from Zedda et al. [67]). (**F**) *Sus scrofa* bone structure: plexiform tissue, osteocytes (2) surrounded by lacunas (image adapted from Zedda [68]).

Table 2 shows a comprehensive comparison of the histological differences between human and non-human bones, supported by Figure 1 that shows histological images of different bone structures. 

**Table 2:** Comparison of the histological differences between human and non-human bones and the significant variations in Haversian canal diameter, osteon diameter, and Haversian canals per mm^2^.

		General Organisation	Central Canal Characteristics	Osteon Characteristics	Density of the Canal	Refs.
Human Bone		Human bone is characterized by a compact organization of osteons that contain a central canal with cellular and vascular elements. Cement lines with well-defined edges and osteocytes in their corresponding lacunas allow for easy identification and delimitation of the osteons.	The mean diameter of the canal is 35.92 mm with a standard deviation of 2.12 mm.	The mean diameter of the osteon is 111.07 mm with a standard deviation of 2.25 mm.	The mean density of the canal per square millimeter is 6.23 with a standard deviation of 0.30.	[58,59]
Non-Human Bone	Hen Bone	Haversian bone exhibits a high density of small-sized osteons per square millimeter, with scarce interstitial systems.	The mean diameter of the canal is 29.49 mm, with a standard deviation of 2.18 mm.	The mean diameter of the osteon is 45.73 mm, with a standard deviation of 4.66 mm.	The density of the canal per square millimeter is 19.36, with a standard deviation of 1.25.	[58,60,61]
	Cat Bone	Cat bone has a high density of medium-sized osteons with a significant number of interstitial systems among them.	The mean canal diameter is 10.60 mm, with a standard deviation of 1.01 mm.	The mean diameter of the osteon is 49.93 mm, with a standard deviation of 3.29 mm.	The mean density of the canal per square millimeter is 8.28, with a standard deviation of 1.63.	[58,62,63]
	Canis Bone	Canis bone is characterized by a high density of medium-sized osteons per square millimeter, with a significant number of interstitial systems among them.	The mean canal diameter is 12.37 mm with a standard deviation of 1.91 mm.	The mean diameter of the osteon is 59.39 mm with a standard deviation of 3.63 mms.	The mean density of the canal per square millimeter is estimated to be 8.75 with a standard deviation of 0.47.	[58,64]
	Bovine Bone	Bovine bone tissue closely resembles human bone tissue upon observation. Its osteons are more oval than human ones, and the borders are not well defined.	The mean canal diameter is 26.24 mm, with a standard deviation of 3.75 mm.	The mean diameter of the osteon is 118.34 mm, with a standard deviation of 16.40 mm.	The canal density per mm^2^ is a mean of 3.65, with a standard deviation of 0.65.	[58,65]
	Pig Bone	The bone of a pig has some distinguishable characteristics when compared to human bone. It has a lower number of osteons per square millimeter. However, these osteons are slightly larger, more irregular, and have less defined borders. Osteocytes are present in the bone, surrounded by their characteristic lacunas.	The mean diameter of the canal is 40.09 mm, with a standard deviation of 14.82 mm.	The mean diameter of the osteon is 114.76 mm, with a standard deviation of 8.19 mm.	The canal density per square millimeter is 1.97, with a standard deviation 0.39.	[58,66]

The number of osteons that various relevant publications deem sufficient for representativeness varies [69]. Cattaneo et al. [7] suggested using just three osteons per specimen. However, Martiniaková et al. [3] and Dominguez and Crowder [50] advocated utilizing a minimum of 50 complete osteons per specimen. A minimum of 25 to 30 undamaged osteons have also been utilized in certain articles on age-at-death calculation in human bone from histomorphometry [70,71]. It is challenging to determine the precise number of osteons required for representativeness; however, it appears that fewer osteons raise the possibility of a misclassification. A single outlier measurement on a small sample of osteons can skew the mean in one direction or another and result in a misclassification. Other factors than the quantity of osteons may have an impact on their representativeness. Both histomorphological and histomorphometric variability was found in a study by Cummaudo and colleagues [28] in various bones of the same individual, in different regions of the same bone, and in different areas of the same section. This heterogeneity may have an impact on the efficiency of histomorphometric techniques and result in inaccurate findings. In other instances, the examined osteons were restricted to a small region that would not have been a good representation of the original bone. Therefore, although more bone surface often equates to more osteons, this is not always the case. The number of osteons examined appears to have a greater bearing on the reliability of histomorphometric methods when applied to fractured bones. The existence of diagnostic change can significantly lower this number, and the observed group of osteons may not be typical due to the random effect of histomorphometric variability.

## 3. Limitations and Challenges

One of the greatest challenges in human histological analysis has been examining exhumed or old skeletal remains. Fragments, especially small ones, can easily be mistaken for wood, stones, metal, or other materials.

### 3.1. Fragmented Samples and Degraded Bone

One limitation of bone identification is the possibility of only having a small fragment of bone to work on, which is often the case in forensic and archaeological samples. If the bone is too small, there is often no chance of being able to identify the bone visually, let alone by sex or age. Many techniques rely on being able to obtain a sample of a good size and are not applicable to small fragments, such as isoelectric focusing for protein analysis. HPLC can be used with small samples, but often, it destroys the sample while dissolving it. DNA analysis is still in its early stages for bone identification, but it is improving and is likely to become the method of choice for identifying human versus non-human bone [72,73]. Despite advancements in the techniques used to identify small and degraded bone samples, the identification of bones still poses a challenge. Further research in this area would be beneficial in improving bone identification methods.

The application of the methods described in this paper to archaeological or surface-recovered material presents a unique set of challenges, particularly when it comes to degraded bones. The success of any histological method is contingent upon the preservation of the material under study, and bone, in particular, is susceptible to a wide range of post-mortem alterations. As bone degradation increases, methods such as thin sectioning become more challenging, as excessive drying out of the material can lead to cracking and breakage of the section. Staining the sections may also prove less effective, as heavily altered bone may not absorb dye evenly, thereby affecting the contrast between mineralized and non-mineralized areas. Light microscopy of degraded bone may reveal a mass of crushed and twisted collagen fibres with no discernible organization into woven and lamellar tissues [73,74,75].

### 3.2. Cross-Reactivity with Animal Species

There have been many reported cases where bone samples, which were previously thought to be from humans and found at ancient sites, were later determined to be from animals after examination. These misidentifications frequently arise from the significant deterioration of the bone material, exacerbated by the analyst’s inability to determine the species of origin. Additionally, hares and rabbits present bones that, despite their smaller size compared to human bones, exhibit similar densities, leading to potential misinterpretations. Moreover, pig bones bear striking resemblances to human bones in both appearance and microscopic structure [76]. The inherent similarities in the physical, chemical, and mechanical properties of non-human and human bones underscore the necessity for accurate testing methodologies to mitigate the risk of erroneous results. 

It is crucial that such tests be conducted with the utmost care to prevent any wrongful accusations. The decision to proceed with destructive physical tests relies on ethical considerations and the balance of benefits and harm to society. As well as the concern for ethical implications, it is often not desirable to use up precious archaeological or forensic material when a similar result can be reached using a non-destructive test.

## 4. Applications of Histomorphological Identification of Human Bones

The ability to identify species origins from bone fragments has potential applications in archaeology, forensic science, and palaeontology. This is particularly the case in regions where human and animal bones have become intermixed, making it difficult to determine whether a bone is of human or animal origin. In forensic science, the identification of human remains in disaster victim identification (DVI) is of particular importance. DVI teams may be required to identify victims following mass fatalities in a number of different scenarios, for example, terrorist incidents, air disasters, or war or natural disasters. In recent years, several papers have highlighted the need for standardization in methods used for the identification of human remains, stressing that techniques used should be both reliable in the degree of accuracy and precision and must be both repeatable and reproducible.

## 5. Conclusions

Since the 1800s, anthropology and archaeology have employed the histomorphological analysis of bone to evaluate species-specific characteristics. The purpose of this study was to evaluate currently used histomorphometric techniques for identifying human versus non-human bone types. This article has highlighted the difficulties involved in positively identifying human bone histologically. Several important aspects of this work should be taken into consideration, such as the discriminant function as it is currently presented, which might benefit from revision in certain techniques due to possible inaccuracies in the original publication. Far from providing a single coherent method, the authors indicated that combining several techniques may be necessary to confirm results. Considering the data from the available literature, it is evident that until further information is obtained, applying such techniques in actual forensic or archaeological cases needs to be done cautiously. The application of histological techniques to archaeology has much potential. Still, it is vital that researchers have a good understanding of histological theory and methods so that they do not make incorrect assumptions about their findings.

## Figures and Tables

**Table 1:** The main findings of the most important morphometric studies.

Study	Bone Analysed	Main Findings	Ref.
Cattaneo et al.	Long bones	Quantitative microscopy and canonical discriminant function accurately identified sample origins, showing superiority over standard microscopy. Proteomics identification was successful, whereas mitochondrial DNA amplification from human bone was unsuccessful. Biomolecular studies yielded no false positives.	[7]
Martiniaková et al.	Femur	Light microscopy examination of Haversian bone distinguished between humans and different species, humans being the only species from the ones included in the study to show dense Haversian tissue.	[3]
Crescimanno and Stout	Humerus, femur, and ribs	Used osteon circularity to distinguish between different species. They reported a non-significant difference between males and females.	[49]
Pirok et al.	Femur and ribs	The study was conducted just on humans and emphasized major differences in the microstructure of the ribs and the femur.	[51]
Pfeiffer	Ribs and femur	Significant differences were reported between the microstructure of the bones from different human populations from 18th, 19th, and 20th centuries.	[52]
